# Spontaneous Remission of Acromegaly After Pituitary Apoplexy in a Middle-Aged Male

**DOI:** 10.31486/toj.20.0002

**Published:** 2021

**Authors:** Sarah Alam, Suraj Kubihal, Alpesh Goyal, Viveka P. Jyotsna

**Affiliations:** Department of Endocrinology and Metabolism, All India Institute of Medical Sciences, New Delhi, India

**Keywords:** *Acromegaly*, *apoplexy*, *headache*, *spontaneous remission*

## Abstract

**Background:** Pituitary apoplexy results from hemorrhage, infarction, or hemorrhagic infarction within a pituitary tumor. Subclinical or clinical apoplexy is not uncommon in acromegaly, owing to the large size of the tumor at initial detection. Growth hormone excess in acromegaly often persists following surgery. However, in rare instances, pituitary apoplexy may present a spontaneous cure to growth hormone excess.

**Case Report:** A 40-year-old male presented with holocranial headache for the past 16 years that had worsened in severity during the prior year. Two months before presentation, he experienced a severe headache that he described as the worst headache of his life. The patient had prominent acromegaloid features that he ignored, as they seemed to cause no harm. The patient had no signs of clinically active disease. Magnetic resonance imaging of the brain revealed a pituitary macroadenoma with evidence of hemorrhage. Serum insulin-like growth factor 1 and oral glucose–suppressed serum growth hormone levels were normal, suggestive of inactive or silent disease. Pituitary apoplexy causing spontaneous remission of acromegaly was diagnosed, and close follow-up was planned for the evolution of hypopituitarism.

**Conclusion:** This case highlights a rare presentation of acromegaly in which an episode of symptomatic pituitary apoplexy revealed the diagnosis of pituitary adenoma and led to the cure of growth hormone hypersecretion.

## INTRODUCTION

Pituitary apoplexy is a rare but potentially fatal clinical syndrome that results from hemorrhage, infarction, or hemorrhagic infarction within a pituitary tumor. Pituitary apoplexy has been described at an incidence of 0.17 episodes per 100,000 patients per year, affecting approximately 2% to 12% of patients with pituitary adenoma.^1^ The clinical presentation is characterized by symptoms of acute onset severe headache in association with vomiting, visual disturbances, cranial nerve palsies, loss of consciousness, and meningismus. However, pituitary apoplexy may be subclinical or asymptomatic in up to one-fourth of patients with pituitary adenoma and is incidentally discovered on imaging or at autopsy.^2-4^

Precipitating factors for pituitary apoplexy include head trauma, major surgery (especially orthopedic and cardiac surgeries), uncontrolled hypertension, diabetes mellitus, anticoagulant use, cranial irradiation, dopamine agonist therapy, and dynamic testing.^5-10^ The etiopathogenesis for development of pituitary apoplexy includes the outgrowth of a pituitary tumor in relation to its blood supply and compression of the pituitary gland vascular supply by the expanding tumor mass, resulting in necrosis and infarction.^11^ Pituitary apoplexy is more often reported with nonfunctioning pituitary adenomas (NFPAs) than with functioning adenomas, an observation attributed to the fact that NFPAs are often diagnosed late when they are already quite large in size.^11,12^ However, the association with NFPAs may be slightly overestimated, as functionality may not be apparent because of the pituitary damage caused by apoplexy.^13^ Pituitary apoplexy has also been described with prolactinomas, corticotropinomas, and growth hormone–producing tumors.

The diagnosis of pituitary apoplexy is established via computed tomography or magnetic resonance imaging (MRI) of the brain demonstrating a pituitary tumor with evidence of necrosis and/or hemorrhage. Historically, pituitary apoplexy was considered a neurosurgical emergency, and surgical decompression was performed in all cases.^3^ However, studies have demonstrated favorable outcomes with conservative management, suggesting that surgical treatment should be reserved for select patients with visual defects and a reduced level of consciousness.^14-17^

Pituitary apoplexy can result in multiple acute endocrine insufficiencies, the most common and clinically significant being secondary adrenal insufficiency.^18,19^ When it is a complication of functional pituitary adenoma, pituitary apoplexy can sometimes (rarely) lead to the resolution of hormonal hypersecretion and remission of the disease state.^20-22^ We describe such an occurrence in a middle-aged male with acromegaly who had spontaneous cure of his growth hormone excess state following an episode of symptomatic pituitary apoplexy.

## CASE REPORT

A 40-year-old male presented to our clinic with a history of holocranial headaches for the past 16 years that had gradually worsened. For the 1 year prior to presentation, he was troubled on an almost daily basis with severe headaches that also caused frequent nocturnal awakenings. He had been treated elsewhere for migraine headaches without significant symptomatic improvement.

He had noticed acromegaloid changes in the form of coarsening facial features and an increase in the size of his hands and feet during the prior 10 years. However, he did not seek medical attention for these changes, as they seemed to cause no harm. The patient had no history of seizures, loss of consciousness, recurrent vomiting, or episodes of transient visual obscurations. He denied a history of hypertension or diabetes mellitus. Two months before the current presentation, he had an episode of severe headache that he described as the worst headache of his life. The episode lasted for 4 to 5 hours, and he was treated with intravenous analgesics and fluids. No nausea, vomiting, dizziness, blurring of vision, diplopia, or any other focal neurologic deficit was associated with the episode. He denied a history of head trauma, anticoagulant use, prior surgical intervention, cranial irradiation, and pituitary dynamic testing.

**Figure 1.**
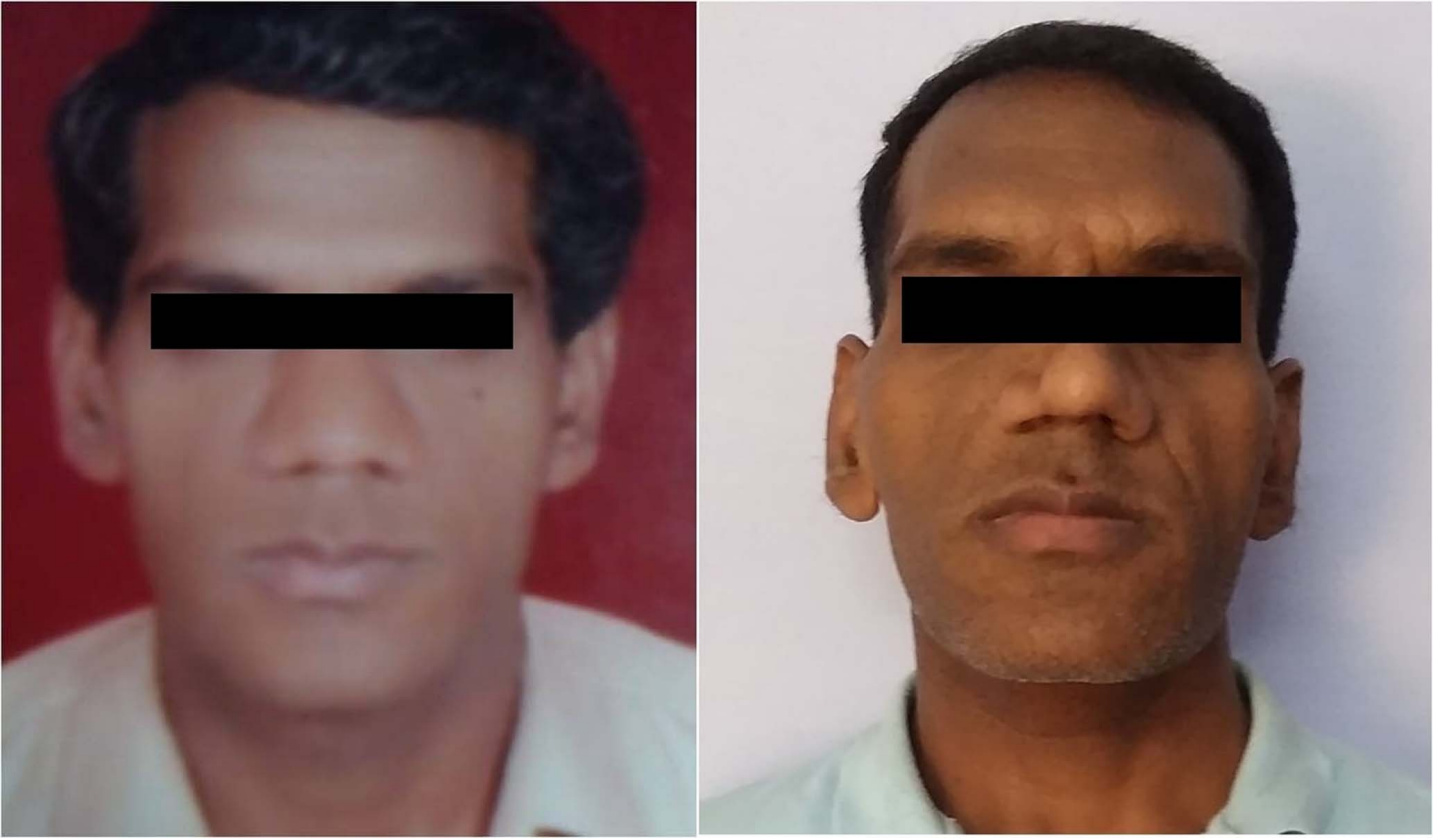
Clinical photographs show facial features of acromegaly in the patient in 2008 (left panel) and in 2019 (right panel).

**Figure 2.**
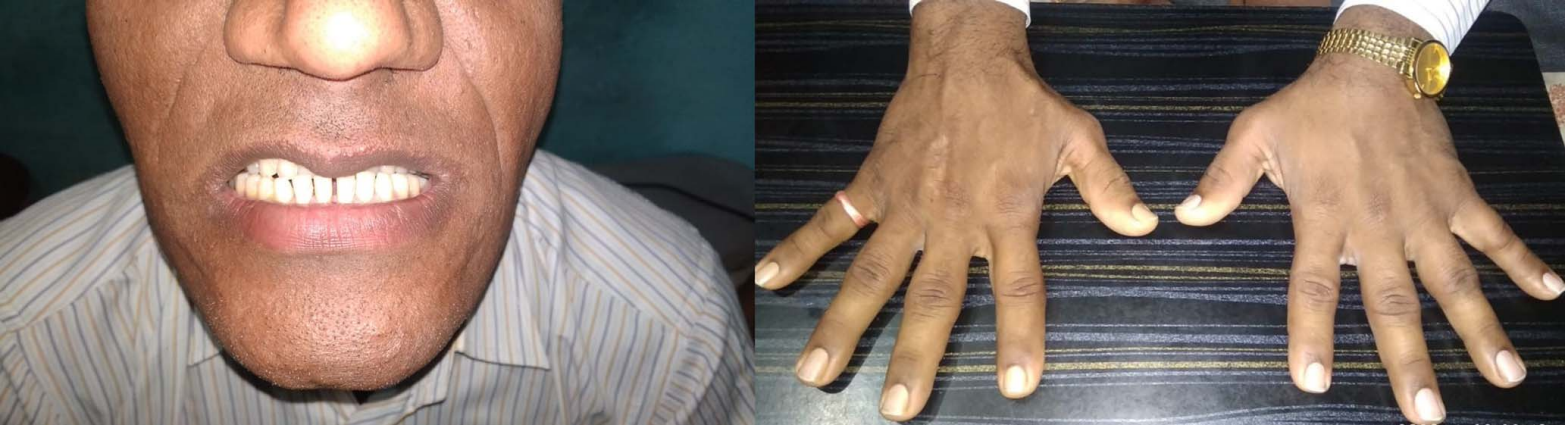
Clinical photographs at admission show dental malocclusion and increased interdental spaces (left panel) and enlarged hands (right panel).

**Figure 3.**
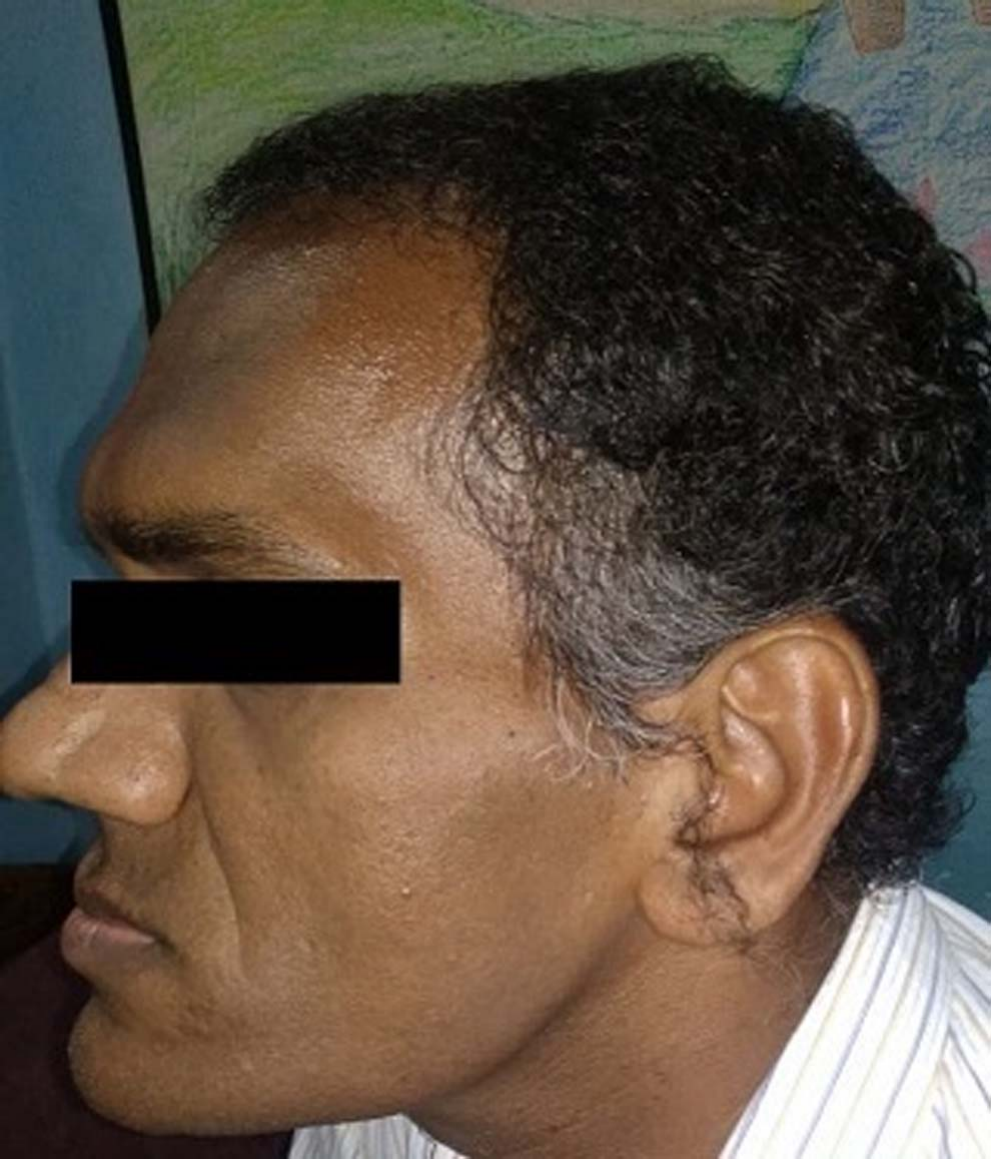
Clinical photograph at admission shows prognathism.

On examination, the patient's pulse rate was 84/min, and blood pressure was 128/76 mmHg. His height, weight, and body mass index were 175 cm, 74 kg, and 24.1 kg/m^2^, respectively. The clinical features of acromegaly were confirmed on physical examination; however, no signs of active disease were noted (Figures 1, 2, and 3). Visual acuity was 6/6 assessed using a Snellen chart, and field testing done by confrontation perimetry was normal. The remainder of the patient's general and systemic examination was unremarkable.

**Figure 4.**
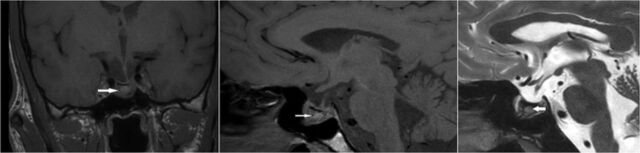
Magnetic resonance imaging of sellar region. T1-weighted coronal (left panel) and sagittal (middle panel) and T2-weighted sagittal (right panel) images show macroadenoma (arrows) with stalk deviation toward the left side. The lesion is hyperintense on T1-weighted images and hypointense on the T2-weighted image, suggestive of hemorrhage within.

Laboratory evaluation (Table) revealed normal complete blood count, liver, and renal function tests. Endocrine profile included normal serum free thyroxine, thyroid-stimulating hormone, 8 am cortisol, testosterone, prolactin, and insulin-like growth factor 1. Growth hormone suppression test with 75 g glucose revealed nadir growth hormone of <1 μg/L (normal). These investigations suggested normal pituitary function with no evidence of growth hormone hypersecretion. MRI showed enlarged pituitary fossa, a 12-mm macroadenoma in the right half of the pituitary gland, and evidence of hemorrhage within the mass (Figure 4).

**Table.** Laboratory Results at Presentation

Parameter	Result	Reference Range
Hemoglobin, g/L	141	120-150
Total leukocyte count, cells/L	6.75 × 10^9^	4-11 × 10^9^
Platelets, cells/L	209 × 10^9^	250-450 × 10^9^
Urea, mmol/L	2.8	3.33-6.66
Creatinine, mmol/L	0.08	0.03-0.11
Uric acid, mmol/L	0.32	0.15-0.39
Calcium, mmol/L	2.27	2.12-2.59
Phosphate, mmol/L	1.55	1.39-1.74
Serum alkaline phosphatase, IU/L	295	240-840
Serum glutamic oxaloacetic transaminase/Serum glutamic pyruvic transaminase, IU/L	19/24	<40/<40
Albumin, g/L	45	35-45
Sodium/Potassium, mmol/L	145/4.7	135-145/3.5-5.5
Total cholesterol, mmol/L	4.18	<5.17
Triglycerides, mmol/L	1.8	<1.70
Low density lipoprotein cholesterol, mmol/L	2.58	<3.36
High density lipoprotein cholesterol, mmol/L	0.77	>1.03
Total thyroxine, nmol/L	141.5	65.6-181.5
Free thyroxine, pmol/L	18.53	11.97-21.88
Thyroid-stimulating hormone, mIU/L	1.27	0.27-4.2
Testosterone, nmol/L	11.19	8.3-28.77
8 am cortisol, nmol/L	293.2	171.0-535.1
Prolactin, μg/L	4.7	4.6-21.4
Insulin-like growth factor 1, μg/L	173.9	88.3-209.9
Growth hormone suppression test, μg/L		
0 min	0.476	Nadir <1 μg/L considered normal.
30 min	0.169	
60 min	0.080	Nadir >1 μg/L suggests growth hormone excess.
90 min	0.143	
120 min	0.064	

The differentials of acute severe headache include subarachnoid hemorrhage, pituitary apoplexy, hypertensive encephalopathy, acute cerebral ischemia, intracranial hypotension (spontaneous or post lumbar puncture), idiopathic intracranial hypertension, posttraumatic headache, substance use or withdrawal-related headache, migraine, cluster headache, and idiopathic thunderclap headache. Our patient had severe acute headache with clinical features suggestive of acromegaly and neuroimaging evidence of pituitary macroadenoma with hemorrhage, suggestive of pituitary apoplexy. However, clinical and biochemical markers of acromegaly activity were normal, suggestive of inactive disease. The patient had no history of transsphenoidal surgery or cranial irradiation. Pituitary apoplexy causing spontaneous remission of acromegaly was therefore diagnosed.

Because the laboratory evaluation did not reveal any endocrine abnormalities, no specific treatment was initiated. The severity of headache reduced significantly during the course of the patient's hospital stay, and he was advised to take oral analgesic paracetamol when needed. He was counseled regarding the need for close follow-up to detect hypopituitarism and was discharged with a plan to repeat pituitary function and neuroimaging at an interval of 3 to 6 months.

## DISCUSSION

The diagnosis of acromegaly is often delayed by many years (average 10 to 12 years) because of the slow and subtle progression of physical findings that patients fail to notice.^23^ In fact, many patients are diagnosed on the appearance of symptomatic mass effects caused by the tumor or incidentally when seeing a physician for some other ailment. The delay in diagnosis exposes patients to the risk of tumor progression and a state of uncontrolled growth hormone hypersecretion, resulting in increased morbidity and mortality. Our patient had had symptoms of growth hormone excess for 10 years, but he ignored them because the symptoms did not result in any functional limitation. However, the symptom of headache prompted him to see multiple physicians who failed to diagnose the primary disease.

In more than 95% of cases of acromegaly, the cause for growth hormone hypersecretion is a somatotroph pituitary adenoma, commonly macroadenoma.^24^ Surgery is the first line of therapy for most patients with acromegaly. However, remission rates are only 80% for patients with microadenoma and <50% for those with macroadenoma,^25^ implying that a large proportion of patients are left with a persistent growth hormone hypersecretion state, requiring consideration for medical therapy (eg, somatostatin analog, pegvisomant, cabergoline), radiation therapy (stereotactic radiosurgery, fractionated radiotherapy), or repeat surgery. Our patient had an undiagnosed pituitary macroadenoma that bled, possibly because of its large size, and resulted in the spontaneous cure of acromegaly without therapeutic intervention.

Clinical remission of acromegaly following apoplexy is extremely rare and to our knowledge has only been reported in 29 cases in the literature.^26-30^ While a precipitating event such as gastrointestinal hemorrhage or gadolinium-DTPA (diethylenetriaminepentaacetic acid) administration causing an acute change in blood pressure was identified by some authors, no trigger could be identified by others.^26,29,30^ Our patient had no recognizable precipitating factor for the acute event.

Pituitary apoplexy is an important cause of hypopituitarism. A literature review reveals that more than two-thirds of patients who had cure of acromegaly following pituitary apoplexy developed other pituitary hormone deficiencies, and a small fraction (<10%) even evolved to panhypopituitarism.^26^ This observation highlights the importance of close surveillance for pituitary function in any patient with suspected or confirmed pituitary apoplexy. While we found no pituitary hormone deficits in our patient at the current presentation, he will be closely followed for potential deficits.

## CONCLUSION

Pituitary apoplexy should be considered in the differential diagnosis of a patient presenting with acute severe headache. In rare instances, pituitary apoplexy may result in a spontaneous cure of the pituitary hypersecretion state. Patients with suspected or confirmed pituitary apoplexy should undergo periodic assessment for the development of hypopituitarism. This case highlights a rare presentation of acromegaly in which an episode of symptomatic pituitary apoplexy revealed the diagnosis of pituitary adenoma and led to the cure of growth hormone hypersecretion.

## Notes

*This article meets the Accreditation Council for Graduate Medical Education and the American Board of Medical Specialties Maintenance of Certification competencies for Patient Care and Medical Knowledge.*
